# Insights into the Interaction of Heart Failure with Preserved Ejection Fraction and Sleep-Disordered Breathing

**DOI:** 10.3390/biomedicines11113038

**Published:** 2023-11-13

**Authors:** Michael Wester, Michael Arzt, Frederick Sinha, Lars Siegfried Maier, Simon Lebek

**Affiliations:** 1 Department of Internal Medicine II, University Hospital Regensburg, 93053 Regensburg, Germany; michael.arzt@ukr.de (M.A.); lars.maier@ukr.de (L.S.M.); 2 Department of Molecular Biology, University of Texas Southwestern Medical Center, Dallas, TX 75390, USA

**Keywords:** heart failure, HFpEF, obstructive sleep apnea, central sleep apnea, pathomechanisms

## Abstract

Heart failure with preserved ejection fraction (HFpEF) is emerging as a widespread disease with global socioeconomic impact. Patients with HFpEF show a dramatically increased morbidity and mortality, and, unfortunately, specific treatment options are limited. This is due to the various etiologies that promote HFpEF development. Indeed, cluster analyses with common HFpEF comorbidities revealed the existence of several HFpEF phenotypes. One especially frequent, yet underappreciated, comorbidity is sleep-disordered breathing (SDB), which is closely intertwined with the development and progression of the “obese HFpEF phenotype”. The following review article aims to provide an overview of the common HFpEF etiologies and phenotypes, especially in the context of SDB. As general HFpEF therapies are often not successful, patient- and phenotype-individualized therapeutic strategies are warranted. Therefore, for the “obese HFpEF phenotype”, a better understanding of the mechanistic parallels between both HFpEF and SDB is required, which may help to identify potential phenotype-individualized therapeutic strategies. Novel technologies like single-cell transcriptomics or CRISPR-Cas9 gene editing further broaden the groundwork for deeper insights into pathomechanisms and precision medicine.

## 1. Introduction

Heart failure with preserved ejection fraction (HFpEF) is a widespread disease with a prevalence of approximately 1% in developed countries and it is expected to rise to more than 5% among elderly populations (i.e., >70 years of age) [1]. The 5-year mortality rates of patients with HFpEF are estimated to be between 55% and 74% [2]. HFpEF symptoms such as dyspnea, fatigue, sleeping difficulties, depression, chest pain [1], and recurrent hospitalizations limit patients’ daily physical and social activities, as well as their capacity to work, thus leading to a poor quality of life [3]. HFpEF causes more than 0.5 million hospitalizations per year in Europe. Notably, hospitalizations contribute to 70–80% of the total health care costs for HFpEF patients, with an average yearly cost of ≈EUR 16,000 per patient [4,5].

These figures and trends are concerning because, in contrast to patients that have heart failure with reduced ejection fraction (HFrEF), currently only one class of pharmacological drugs has been shown to reduce morbidity in patients with HFpEF [1]. Therefore, attention has been redirected to lifestyle interventions such as exercise training [6,7] and the treatment of comorbidities [8] in order to prevent the progression of HFpEF and to reduce patient symptom burdens.

One such comorbidity is sleep-disordered breathing (SDB), which affects up to 58% (or up to 80% in certain cohorts) of HFpEF patients [9,10,11]. SDB presents in HFpEF patients either as predominantly obstructive sleep apnea (OSA) or as predominantly central sleep apnea (CSA) [9]. Treatment of OSA in patients with HFpEF provides an opportunity through which to improve quality of life [12] and exercise capacity [13], and it has the potential to prevent the progression of HFpEF via reductions in the arterial blood pressure and cardiac workload, as well prevention of cardiac remodeling [14,15]. Besides these, therapeutic strategies for patients with HFpEF and SDB are limited, especially with respect to pharmacological interventions. This highlights the need for a better understanding of the presence and effects, as well as its treatment, of SDB in patients with HFpEF.

The aim of this review article is to summarize the up-to-date evidence on HFpEF and SDB to provide an overview of the intricate relationship between both diseases, as well as to identify important gaps in the knowledge and research needs, which might ultimately lead to improved and patient-individualized therapeutic strategies for HFpEF patients with SDB.

## 2. Phenotypes and Symptoms of HFpEF and SDB

### 2.1. Epidemiology and Diagnosis of HFpEF

Left ventricular diastolic dysfunction, which is a precursor to HFpEF [16], is highly prevalent in asymptomatic community samples, and it affects almost one third of adults aged above 45 years [17]. As increasing age is a major risk factor [1] in the prevalence of diastolic dysfunction, and, as the prevalence of HFpEF is higher in the elderly, nearly half of all patients with heart failure (HF) have a preserved ejection fraction [1], which reaches to 65–77% in patients that are ≥67 years of age [18]. In absolute numbers, women outnumber men (ratio ≈ 2:1) [19]. However, this imbalance is, in part, caused by the higher life expectancy of women and the lower risk of death after the diagnosis of HFpEF; however, this discrepancy is alleviated after accounting for age and other risk factors [20].

Patients with HFpEF have a high rate of recurrent hospitalizations. After an episode of acute HF, the 30 day all-cause readmission rate is up to 21% and the HF-specific readmission rate up to 10%, thus reflecting the large burden of comorbidities in HFpEF patients [21]. The readmission rates for one year are up to 63% and 37% for all-cause and HF-specific readmissions, respectively [21]. The high rates for hospitalizations cause significant costs for the health care systems. Annual costs have been estimated to be up to USD 27,000 (in the USA) [22] or EUR 16,000 (in the EU) [5] per patient. The high prevalence and morbidity of HFpEF impose a significant burden on public health care.

Based on ejection fraction (EF), patients with chronic HF are classified as having HFpEF (EF ≥ 50%), heart failure with mildly reduced ejection fraction (HFmrEF, EF 40–49%), or HfrEF (EF < 40%) according to current European guidelines and position papers [1,23,24].

HFpEF is a clinical syndrome composed of many different etiologies, which complicates the aim of establishing a definition of clear diagnostic criteria [1]. The current guidelines of the European Society of Cardiology (ESC) define HFpEF as the combination of (1) the presence of symptoms and signs of HF (e.g., dyspnea, ankle swelling, and elevated jugular venous pressure), (2) a left ventricular ejection fraction of ≥50%, and (3) elevated levels of natriuretic peptides (BNP ≥ 35 pg/mL and/or NT-pro-BNP > 125 pg/mL) plus structural (left ventricular hypertrophy, left atrial enlargement, etc.) or functional (diastolic dysfunction) heart disease [1].

Also, there are empirically derived scoring systems for the diagnosis of HFpEF. Similar to their guideline criteria, the HFA-PEFF algorithm (Heart Failure Association, and PEFF stands for the steps of diagnostic work up, i.e., pretest assessment, echocardiographic and natriuretic peptide score, functional testing, and final etiology) [23] and the H2PEF score [25] both require normal left ventricular EF, signs or symptoms of HF, structural cardiac remodeling, signs of diastolic dysfunction, and biomarkers (e.g., abnormal brain natriuretic peptide). For patients with intermediate score values, echocardiographic stress testing (i.e., volume challenge or supine exercise) or invasive testing (right heart catheterization) can be added.

### 2.2. Epidemiology and Diagnosis of SDB

There are two main SDB sub-types that are diagnosed by poly(somno)graphy: central sleep apnea (CSA) and obstructive sleep apnea (OSA) [26]. Mechanistically, in CSA, the central respiratory signal pauses, thereby decreasing or ceasing airflow for ≥10 s [27,28,29]. In patients with OSA, a partial or complete collapse of the upper airway reduces the airflow (hypopnea), or it may even lead to a complete cessation of airflow (apnea) for ≥10 s [29]. An apnea-hypopnea index (AHI) of ≥5 events/h with characteristic symptoms (e.g., witnessed apneas, daytime sleepiness, and snoring) or an AHI of ≥15 events/h (regardless of symptoms) defines OSA [29,30]. The classification of SDB into CSA or OSA is based on the predominant (i.e., ≥50%) type of apneas/hypopneas [27,28,29]. CSA and OSA can occur separately, but could also occur concurrently within the same patient [27,28,29]. Patients with cardiovascular disease, particularly those with central sleep apnea, are less likely to exhibit classic SDB symptoms (which further complicates diagnoses [27,28,29,31,32]).

SDB is a widespread disease, and it currently affects about one billion individuals worldwide, as well as up to 40% in patients with cardiovascular disease [28,33]. In patients with HF (both HFpEF and HFrEF), the prevalence of SDB increases to 50% [9,10]. Notably, HF is associated with a high occurrence of CSA [9,27], whereas the severity of CSA is related to cardiac function.

### 2.3. Phenotypes of HFpEF—Comorbidities and Cluster Analyses

Ever since HFpEF was acknowledged as a distinct clinical syndrome, it has challenged cardiologists with respect to successfully finding definitive diagnostic criteria and offering effective treatment strategies. The difficulties and failures at this task most likely stem from the diversity of underlying etiologies, pathomechanisms, and relevant comorbidities, which defy a diagnostic or therapeutic “one-size-fits-all” approach [18].

Similar to SDB, HFpEF is strongly associated with several frequent diseases and pathologies such as metabolic syndrome (e.g., diabetes, obesity, and hypertension), pulmonary diseases (e.g., COPD and pulmonary hypertension), coronary artery disease, and chronic kidney disease [1,34]. A sedentary lifestyle and insufficient physical activity denominate a common risk factor for metabolic syndrome, arterial hypertension, and HFpEF [35]. Accordingly, increased physical activity has been shown to be among the few therapeutic strategies through which to realistically improve symptomatic outcomes in patients with HFpEF [6]. All of these comorbidities, specifically HFpEF and SDB, share many pathomechanisms that are closely intertwined and may often reciprocally reinforce their detrimental effects. Therefore, the treatment of those comorbidities may be crucial for avoiding the propagation of HFpEF.

Since the first attempt to phenotype HFpEF, many of the different approaches that utilize machine learning have identified distinct HFpEF phenotypes [36]. Based on clinical considerations, four phenotypes can be discerned: the “aging phenotype”, the “pulmonary hypertension phenotype”, the “coronary artery disease phenotype”, and the “obese phenotype” [37]. This clinical classification has been affirmed and specified by novel artificial intelligence-based deep-learning approaches that are based on clinical evaluation, echocardiographic, ECG, laboratory, and proteomic data [18,36,38] (the reviews of [39,40,41] provide an excellent overview).

**Figure 1:**
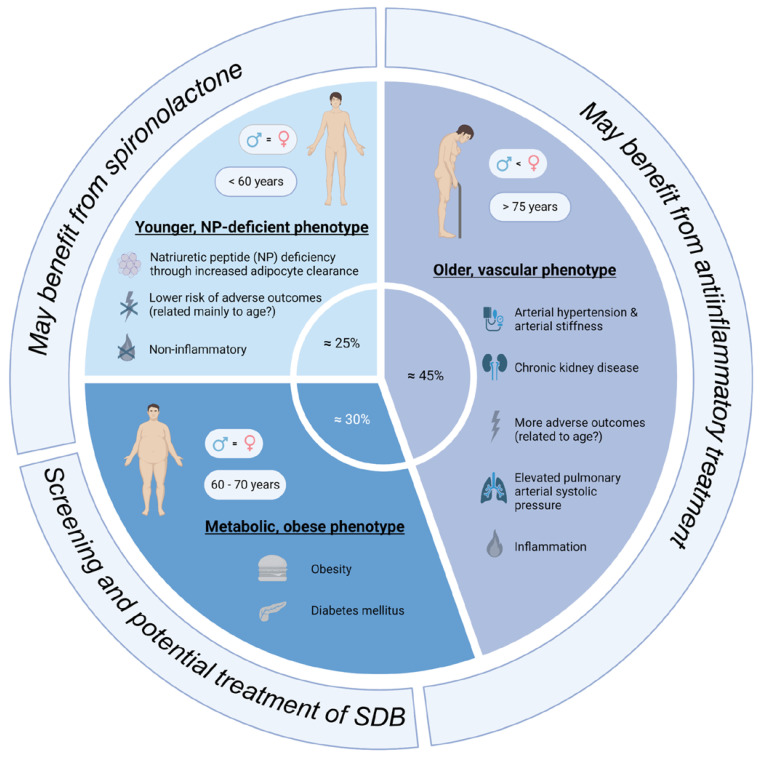
Overview of the main subtypes of HFpEF patients (created with biorender.com, accessed on 23 October 2023).

The review by Peters et al. summarizes three main phenotypes: the “older, vascular aging phenotype”, the “metabolic, obese phenotype”, and the “relatively young, natriuretic peptide deficiency phenotype” (Figure 1) [41]. It is important to acknowledge that these phenotypes cannot be unequivocally discriminated as features of these phenotypes often overlap, and it is not yet clear if these phenotypes can develop into each other. The “older, vascular aging” phenotype accounts for approximately 30–50% of HFpEF patients. Its characteristics are higher age (>75 years), chronic kidney disease, arterial hypertension (together with arterial stiffness), and a high rate of adverse outcomes [41]. These patients often have elevated pulmonary arterial systolic pressure, as well as left atrial and/or right ventricular dysfunction. It seems that systemic inflammation is prevalent in these patients. The “metabolic, obese” phenotype accounts for approximately 25–30% of HFpEF patients. These patients are slightly younger than the “older” phenotype (60–70 years). These patients are obese and often have diabetes mellitus [41]. Epicardial adipose tissue may mechanistically favor HFpEF development in these patients. The comorbidities promote systemic inflammation, which is a contributing factor for HFpEF development. The last phenotype is the “relatively younger, natriuretic peptide deficiency” phenotype, which accounts for approximately 40–45% of HFpEF patients. These patients are relatively young (around 60 years), and they exhibit lower BNP/NT-pro-BNP levels due to increased adipose clearance. The absence of inflammation distinguishes this phenotype from the “metabolic, obese” phenotype. These patients have the lowest risk for adverse outcomes [41].

A cluster analysis of the TOPCAT study cohort also revealed three different phenotypes [42]. However, while the total HFpEF study cohort of TOPCAT did not benefit from treatment with spironolactone, a machine learning approach identified one HFpEF phenotype that, in actuality, showed an improved survival rate [42]. This phenotype was very similar to the “obese phenotype” [37] and the “natriuretic peptide deficiency syndrome” [18], which is highly reminiscent of typical SDB patients (even though SDB was not explicitly mentioned). This “obese phenotype” is characterized by the metabolic syndrome, which increases arterial stiffness, promotes systemic inflammation, and activates the sympathetic nervous system [37]. The most common comorbidities of this phenotype are obstructive sleep apnea, diabetes mellitus, and chronic kidney disease [37]. The prevalence of the “obese phenotype” in the TOPCAT study cohort was 31% [42]. SDB is highly prevalent in obese (40–60%) compared to non-obese (10–20%) HFpEF patients [37,43], which highlights the importance of SDB as a potentially treatable and modifiable comorbidity in HFpEF.

## 3. Treatment Options for HFpEF and SDB

### 3.1. General Treatment Options for Patients with HFpEF

While there have been major advances in the treatment of HFrEF, pharmacological treatment options for HFpEF remain limited [1,24]. The recent consensus statement of the ESC on phenotyping in patients with HFpEF provides an excellent overview on phenotypes, comorbidities, as well as on specific and emerging treatment options [44]. As the activation of the renin-angiotensin-aldosterone system (RAAS) causes myocardial fibrosis and endothelial dysfunction, which are both hallmarks of HFpEF, RAAS inhibition is thus expected to be beneficial in HFpEF patients [45]. However, neither angiotensin-converting enzyme (ACE) inhibitors [46,47,48], aldosterone receptor antagonists (i.e., spironolactone) [49], or the angiotensin-receptor neprilysin inhibitors [50] showed a reduction in cardiovascular death or hospitalizations. Also, beta blockers, which are a mainstay of HFrEF therapy, failed in reducing mortality in HFpEF patients [51]. Only sodium-glucose cotransporter 2 (SGLT2) inhibitors have been shown to reduce heart failure hospitalizations [24,52,53]. The precise cardiac mechanisms of SGLT2 inhibitors are not yet completely understood, but it has been shown that they inhibit CaMKII activity and pathological CaMKII-dependent signaling [54]. Plus, it seems that they exhibit effects that are especially beneficial in patients with SDB [55]. SGLT2 inhibitors improve renal function, thereby potentially ameliorating the detrimental bidirectional impairment of renal and cardiac function [55]. SGLT2 inhibitors also reduce visceral and subcutaneous adipose tissue, which is also a risk factor for endothelial dysfunction and is a common feature in HFpEF [55].

A novel and emerging type of therapy, especially for the “obese HFpEF phenotype”, may be treatments with glucagon-like peptide-1 (GLP-1) receptor agonists. These drugs have been shown to effectively reduce body weight and blood glucose levels [56,57]. As obesity and diabetes mellitus are prominent features of the obese HFpEF phenotype, this treatment regimen may prove beneficial in these patients, especially as the severity of SDB (specifically in OSA patients) is expected to be ameliorated by weight loss. However, clinical trials on cardiovascular outcomes are still required.

As many common comorbidities have reciprocal detrimental effects on HFpEF, the additional treatment of comorbidities and the optimization of risk factors is pivotal [1]. Active atrial contraction becomes relatively more important for maintaining cardiac filling when the passive diastolic filling of ventricles is impaired. Indeed, a sub-study of the CABANA trial has shown that the ablation therapy of atrial fibrillation reduces mortality and improves quality of life in symptomatic patients, of which the majority (about 75%) had an ejection fraction of >50% [58]. A prospective randomized trial has recently demonstrated reduced pulmonary wedge pressure, improved peak oxygen uptake, and an improved quality of life in patients with HFpEF after obtaining rhythm control with ablation therapy when compared to optimal pharmacological treatment [59]. Exercise training has been shown to improve exercise capacity and diastolic function in patients with HFpEF [6,7]. As both diabetes and arterial hypertension contribute to the development of diastolic dysfunction and HFpEF, an optimal treatment for improving diastolic function and preventing further disease progression is warranted.

Overall, effective pharmacological and non-pharmacological treatment options remain limited for patients with HFpEF. Many different etiologies, pathomechanisms, and comorbidities contribute to this syndrome in which an individualized treatment approach that targets personal risk factors and disease constellations, as well as a discussion of lifestyle optimization with the patient, are warranted.

### 3.2. Specific Treatment Options for Patients with HFpEF and SDB

As specific therapeutic strategies for patients with HFpEF are scarce, the treatment of risk factors and comorbidities such as SDB becomes imperative, especially due to the fact that the “obese HFpEF phenotype” closely resembles typical OSA patients. However, phenotyping studies [39,40,41], as well as the ESC consensus statement [44], do not include SDB as a risk factor, which seems to be an important gap in our knowledge and should be addressed in future analyses. A phenotyping approach of patients with SDB and heart failure has shown that older hypoxic obese patients with HFpEF represent the patient group that may best profit from ASV therapy [60,61].

Apart from lifestyle changes (such as, for example, reduction in alcohol intake, weight loss, etc.), the treatment of SDB consists in delivering positive airway pressure (PAP) therapy. There are three modalities: continuous PAP (CPAP), bilevel PAP (BiPAP), and adaptive servo-ventilation (ASV) [62,63,64]. The vast majority of trials on PAP therapy in HF included patients with HFrEF [65]. One small trial investigated ASV therapy in patients with HFpEF (EF > 50%, AHI > 15/h, n = 36) [66]. ASV therapy improved diastolic function, reduced BNP levels, as well as reduced the composite endpoint of cardiac death and worsening HF [66]. An observational study in patients with HF and SDB found that older obese HFpEF patients were the individuals with the greatest reduction in cardiovascular mortality and morbidity through ASV therapy [61]. These patients also had the best ASV therapy adherence.

Apart from this small but informative study, a recent meta-analysis showed that PAP therapy moderately reduces arterial blood pressure, which is an important risk factor for HFpEF [15]. However, these patients did not have overt heart disease, and it can be speculated that the effects become larger and more clinically relevant in patients with overt heart disease. It can be speculated that the treatment of OSA prevents the progression of HFpEF by reducing risk factors and comorbidities such as arterial hypertension and cardiac workload, thus averting cardiac remodeling [14,15]. To date, the data from large-scale clinical trials are lacking and urgently warranted. Plus, developing new and specific pharmacological strategies for patients with HFpEF and SDB would broaden and improve the spectrum of therapeutic strategies, but this requires a deep understanding of the molecular pathomechanisms.

## 4. Pathophysiological Interactions between SDB and HF

Several similar pathological aberrations are found in SDB and HF, thereby suggesting a mechanistic overlap or even causal interaction between both disorders [27,67]. Even though only limited evidence directly elucidates the mechanisms of diastolic dysfunction in patients with SDB, it is known that many features of SDB, like intermittent hypoxia/reoxygenation, trigger pathological remodeling that might ultimately result in HFpEF [27,67]. For example, patients with an acute myocardial infarction and concomitant SDB more frequently develop diastolic dysfunction, whereas diastolic function does not change when SDB is absent [68,69].

### 4.1. Increased Cardiac Afterload in SDB

Intermittent hypoxia/reoxygenation and arousals with sudden awakening are key features of SDB, thereby leading to an increased production of reactive oxygen species (ROS) and increased sympathetic activation, respectively [15]. As is especially the case in CSA, sympathetic overactivation is promoted by the hypoxia-/hypercapnia-induced stimulation of central and peripheral chemoreceptors, as well as by the deactivation of pulmonary stretch receptors [70]. These mechanisms favor the development of arterial hypertension with an increased cardiac afterload, the subsequent ventricular hypertrophy, and possibly HFpEF [15,67]. Moreover, OSA is related to intrathoracic pressure swings; this significantly increases cardiac wall stress, which also favors hypertrophy and fibrosis [71]. This is highlighted by the observation that OSA, but not CSA, caused an impaired ventricular remodeling in patients after acute myocardial infarction [14].

### 4.2. Inflammation and Structural Remodeling in SDB

Intermittent hypoxia/reoxygenation is also a strong inducer of systemic inflammatory signaling (for example, NF-ΚB-dependent pathways [72,73]). In a rat model of chronic intermittent hypoxia/reoxygenation, the myocardial levels of inflammation markers (e.g., tumor necrosis factor-α and interleukin-6) correlated with myocardial hypertrophy. In addition, the interleukin 6-related MEK5-ERK5 and STAT-3 pathways, which have been linked to myocardial remodeling, were increased after intermittent hypoxia/reoxygenation [73,74]. Chronic intermittent hypoxia/reoxygenation also increases the cardiac expression of matrix metallopeptidase 2, thus leading to fibrosis and subsequent increases in the passive stiffness of the left ventricular extracellular matrix [73,75,76]. In patients with HFpEF, hypoxia increases red cell distribution width, which is an indicator for subclinical inflammation [77].

In addition, intermittent hypoxia/reoxygenation also increases the levels of angiotensin II, which is known to induce myocardial hypertrophy and fibrosis, and it is also associated with HFpEF development [67,78]. As described above, there are several clinical HFpEF phenotypes, and women display HFpEF more frequently than HFrEF, which also indicates a relevant sex-dependent difference to be at play [37,38,39,40]. Indeed, we have recently found decreased myocardial protein levels of the angiotensin II cleavage enzyme ACE2 in women with SDB, which has been associated with an increased frequency of HFpEF [79]. Mechanistically, reduced angiotensin II cleavage means a boost of this signaling cascade, and thus of increased pathological remodeling [79].

### 4.3. Functional Myocardial Remodeling in SDB

**Figure 2:**
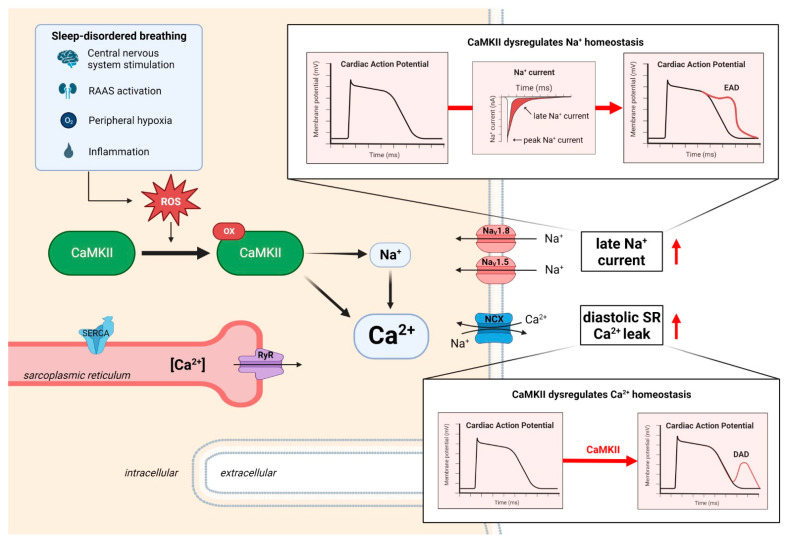
Overview of the CaMKII-dependent dysregulation of myocardial function in SDB that favors the development and progression of diastolic dysfunction. Abbreviations: EAD—early afterdepolarization, DAD—delayed afterdepolarization, NCX—Na^+^/Ca^2+^ exchanger, RAAS—renin–angiotensin–aldosterone system, ROS—reactive oxygen species, and SR—sarcoplasmic reticulum. Created with biorender.com accessed on 23 October 2023.

Besides inflammation and structural remodeling, there is growing evidence that functional myocardial remodeling is also critical for HFpEF development [67,80]. Ca^2+^/calmodulin-dependent protein kinase II (CaMKII) is a central regulator of myocardial function and signaling, and it has also been implemented into the pathomechanisms of SDB [81,82,83] (Figure 2). Thus, CaMKII might be especially important for the “obese HFpEF phenotype” with SDB. While CaMKII controls the excitation–contraction coupling, cellular Ca^2+^ cycling, and the cardiac transcriptome upon physiological homeostasis, overactivated CaMKII is also a key indicator and inducer of various cardiac diseases like HF [81,82]. Dysregulated and chronically overactivated CaMKII signaling has been linked to impaired excitation-contraction coupling, arrhythmias, dysregulated Na^+^ and Ca^2+^ homeostasis, transcriptional changes, inflammation, apoptosis, and fibrosis [81,82,83,84,85,86,87]. Notably, these mechanisms have independently been shown to promote diastolic dysfunction and HFpEF [27,67,80].

Several clinical features of SDB have been shown to possibly promote CaMKII overactivation. Potential mechanisms are an increased cardiac wall stress (e.g., following intrathoracic pressure alterations due to airway obstruction), increased sympathetic activation (e.g., arousals) or oxidative stress (e.g., following cyclic episodes of hypoxemia/reoxygenation) [27,88,89]. Indeed, we recently found an increased ROS production in the atrial cardiomyocytes isolated from patients with SDB, which resulted in a pathologically increased CaMKII activation in the myocardium from patients with SDB [83]. As CaMKII is a central regulator of cardiac Na^+^ and Ca^2+^ homeostasis, CaMKII hyperactivation resulted in an enhanced late Na^+^ current, as well as in an increased diastolic sarcoplasmic reticulum (SR) Ca^2+^ leak [83]. This proarrhythmic dysregulation of cellular ion homeostasis induced early and delayed afterdepolarizations (EADs and DADs) on the cellular level, which subsequently triggered multicellular arrhythmias in the myocardium from patients with SDB [83]. CaMKII-dependent signaling has further been linked to the neuronal Na^+^ channel Na_V_1.8 [90]. Indeed, we found an increased Na_V_1.8 expression in the atrial myocardium from patients with SDB, which further enhanced a late Na^+^ current, as well as subsequently increased the diastolic SR Ca^2+^ leak [91]. All of these CaMKII-dependent proarrhythmic mechanisms found in SDB may eventually result in atrial dysfunction and arrhythmias, thereby promoting HFpEF development.

Importantly, these CaMKII-dependent proarrhythmic aberrations in SDB could be blocked by inhibiting CaMKII, thus making this enzyme a promising therapeutic target in SDB [83,90]. Unfortunately, current compound-based CaMKII inhibitory strategies face several challenges and limitations (e.g., specificity, CaMKII inhibition in organs other than the heart, poor bioavailability, etc.), which currently precludes clinical translation of such a drug [82,92].

### 4.4. Insulin Resistance and Hyperinsulinemia in SDB

SDB, especially OSA, is frequently associated with diabetes mellitus. The prevalence of OSA in patients with type 2 diabetes varies from 18% in primary care [93] to 58% in older individuals [94]. In obese patients with type 2 diabetes, the prevalence of OSA is up to 86% [95]. The interrelationship between SDB and diabetes has been shown to be independent of several clinical covariates, thus suggesting a mechanistic link [96,97]. A recent meta-analysis estimated a 63% increase in incident diabetes in patients with moderate to severe OSA [98]. Vice versa, there have also been studies showing that type 2 diabetes is an independent risk factor for SDB, which can potentially occur by affecting the central and autonomic nervous system [94].

Mechanistically, intermittent hypoxia/reoxygenation, as a key feature of SDB, seems to be critical. Intermittent hypoxia/reoxygenation for 12 weeks impaired glucose tolerance and increased fasting serum insulin levels in leptin-deficient obese mice [99]. Similar observations were made in lean C57BL6/J mice, where intermittent hypoxia/reoxygenation for 14 days increased fasting glucose levels by 67% and impaired glucose tolerance by 27% [100]. The authors further found an impaired insulin sensitivity and pancreatic β-cell function, increased liver glycogen and glucose output, as well as increased oxidative stress in the pancreas—all of them following intermittent hypoxia [100].

Diabetes mellitus is an important risk factor for numerous cardiovascular diseases, including heart failure (both HFpEF and HFrEF), coronary artery heart disease, kidney dysfunction, strokes, etc. [101]. On a cellular level, increased glucose levels and hyperinsulinemia have been shown to induce myocardial hypertrophy, inflammation, and fibrosis [102,103]. Notably, all these mechanisms promote HFpEF development (see above) [67].

### 4.5. Mechanistic Parallels between SDB and HFpEF

The SDB-related mechanisms described above have also been linked to the development of HFpEF, thus indicating a close mechanistic interrelationship [67]. An increased cardiac afterload with myocardial hypertrophy and a disturbed renin-angiotensin-aldosterone system with systemic inflammation, fibrosis, and the subsequent myocardial stiffness have been shown to be key features in HFpEF [67]. As CaMKII is mechanistically involved in hypertrophy and HF, the cardiac specific knock-out of CaMKII is attenuated with afterload-induced cardiac fibrosis, hypertrophy, and the subsequent transition to HF in mice [104,105]. Similar observations were made in a recent study, where a mouse model that was rendered resistant to CaMKII autophosphorylation and its subsequent hyperactivation was protected from afterload-induced hypertrophy, fibrosis, and HF [106]. Afterload-induced CaMKII activation spawns a vicious cycle. This is because CaMKII also increases the inflammatory gene expression (e.g., NF-ΚB) and the activation of the NOD-like receptor pyrin domain containing protein 3 inflammasome in murine cardiomyocytes [107]. Most recently, Kolijn and colleagues found increased markers of oxidative stress (hydrogen peroxide) and inflammation (tumor necrosis factor-α and interleukin-6), as well as increased CaMKII activity, in the left ventricular myocardium of patients with HFpEF [108]. Accordingly, the cardiac overexpression of CaMKII was found to enhance late Na^+^ currents, and it subsequently impaired the diastolic function in mice [109]. As observed in the myocardial biopsies of patients with SDB, the myocardial Na_V_1.8 expression was also increased in patients with cardiac hypertrophy with preserved ejection fraction, whereby HFpEF development was possibly favored by dysregulating cellular Na^+^ and Ca^2+^ homeostasis [91,110]. Further parallels have been found with respect to structural remodeling as the myocardial downregulation of the gap junction protein connexin-43 has been found in both SDB and HFpEF [111,112]. In addition, SDB increases the risk for other disorders as for diabetes mellitus (see above) [98]. Diabetes may further promote HFpEF development as it may possibly enter a deleterious vicious cycle [67,102,103].

However, detailed knowledge about the mechanistic interaction of SDB and HFpEF is still limited. Mechanistic models are difficult to fathom when the HFpEF syndrome is an amalgam of markedly different etiologies and phenotypes. Reductionist disease models are needed to investigate pathomechanisms. Therefore, our group has recently established a novel SDB mouse model by injecting polytetrafluoroethylene (PTFE) into the murine tongue [87]. PTFE is an inert substance that permanently enlarges the murine tongue, which results in spontaneous obstructive apneas, as well as inspiratory flow limitations that subsequently induce increased hypoxia and myocardial ROS production [87,113]. The frequency of apneas correlated with both heart weight (a surrogate for cardiac hypertrophy) and the severity of diastolic dysfunction (E/e’) suggests a causal relationship [87]. We found a pathological dysregulation of myocardial Ca^2+^ homeostasis and proarrhythmic events in SDB mice [113]. Notably, SDB mice, where the oxidative activation sites of CaMKII were ablated in the germline, were protected from cellular Ca^2+^ alterations and arrythmias [113]. This SDB mouse model, therefore, offers the opportunity to specifically study the pathomechanisms connecting the OSA subtype and HFpEF, as well as allows one to optimize and deploy new therapeutic strategies.

## 5. Conclusions and Future Perspectives

SDB and HFpEF are often closely interwoven, and there might even be bidirectional associations that promote a vicious circle. It is important to acknowledge that HFpEF is not a singular syndrome, but that it comprises many different etiologies that result in different phenotypes. There are various underlying pathomechanisms requiring specific prevention and treatment strategies. SDB is a very prevalent disorder with profound implications on the development of cardiovascular disease, especially HFpEF. It is yet unknown whether the severity of SDB is correlated with the severity of diastolic dysfunction or HFpEF. Patients with HFpEF should be regularly evaluated for SDB, which includes the specific history taking regarding signs and symptoms of SDB. As the detection of SDB with polysomnography is cost- and time-intensive, a widespread screening for SDB is currently not feasible. However, the implementation of wearable and artificial intelligence-based devices for screening of SDB is an interesting and growing field in development and research [114,115] that warrants further exploration and scientific validation. If indicated, treatment of SDB should be initiated and followed up closely to ensure optimal therapy adherence. As pharmacological therapy is limited, the treatment of risk factors and comorbidities is even more important. The potential of PAP treatment for SDB in HFpEF patients is still unknown as specific prospective and randomized trials are still missing. This is an important clinical gap of knowledge that should be addressed given the wide prevalence of both SDB and HFpEF, as well as their subsequent symptoms and disease burdens.

For patient-individualized therapies, a more detailed understanding of the various HFpEF entities is urgently needed. Only specific mechanistic insights into each etiology enable the development of targeted efficient therapies. Novel cutting-edge technologies like single-nucleus RNA sequencing allow for transcriptional analyses of individual cell types at a very high resolution, thereby providing detailed insights into the cellular mechanisms of SDB and HFpEF [116]. Moreover, CRISPR-Cas9 gene editing technology has revolutionized the spectrum of therapeutic possibilities. We have recently developed a gene editing strategy to ablate the oxidative activation sites of CaMKII in adult mice in vivo, which subsequently confers a sustained cardioprotection [85]. We also found that *CaMKII*-edited human cardiomyocytes showed preserved diastolic Ca^2+^ levels following hypoxia/reoxygenation, which could possibly improve diastolic cardiac function [85]. As CaMKII activity was increased in SDB and *CaMKII* editing, which attenuated a myocardial remodeling like hypertrophy and fibrosis, this strategy could be beneficial for the “obese HFpEF phenotype” with SDB; however, this remains to be tested [83,106,113]. CRISPR-Cas9 gene editing could also be used to disrupt other pathological signaling cascades that are implemented in other HFpEF phenotypes in the setting of a patient-individualized therapy. Besides CaMKII, there are certainly other targetable effectors that are also involved in the pathogenesis of the various HFpEF phenotypes. Thus, it would be important to further consider, analyze, and modulate the other players that have been implemented in the pathomechanisms in order to develop new therapeutic concepts. Future animal studies are needed that actually test whether targeting the above-described mechanisms confers cardioprotection in HFpEF in vivo, which could be a steppingstone toward a potential clinical trial, as well as toward new and advanced therapeutic strategies.

## Data Availability

No new data were created or analyzed in this study. Data sharing is not applicable to this article.
